# Evaluation of the Alveolar Crest and Cemento-Enamel Junction in Periodontitis Using Object Detection on Periapical Radiographs

**DOI:** 10.3390/diagnostics14151687

**Published:** 2024-08-04

**Authors:** Tai-Jung Lin, Yi-Cheng Mao, Yuan-Jin Lin, Chin-Hao Liang, Yi-Qing He, Yun-Chen Hsu, Shih-Lun Chen, Tsung-Yi Chen, Chiung-An Chen, Kuo-Chen Li, Patricia Angela R. Abu

**Affiliations:** 1 Department of Periodontics, Division of Dentistry, Taoyuan Chang Gung Memorial Hospital, Taoyuan City 333423, Taiwan; an840802@cgmh.org.tw; 2 Department of Operative Dentistry, Taoyuan Chang Gung Memorial Hospital, Taoyuan City 333423, Taiwan; louiszzzzz@cgmh.org.tw; 3 Department of Program on Semiconductor Manufacturing Technology, Academy of Innovative Semiconductor and Sustainable Manufacturing, National Cheng Kung University, Tainan City 701401, Taiwan; m28121562@gs.ncku.edu.tw; 4 Department of Electronic Engineering, Chung Yuan Christian University, Taoyuan City 320234, Taiwan; s11126308@cycu.edu.tw (C.-H.L.); s11126307@cycu.edu.tw (Y.-Q.H.); s11126306@cycu.edu.tw (Y.-C.H.); 5 Department of Electronic Engineering, Feng Chia University, Taichung City 407301, Taiwan; tsungychen@fcu.edu.tw; 6 Department of Electrical Engineering, Ming Chi University of Technology, New Taipei City 243303, Taiwan; 7 Department of Information Management, Chung Yuan Christian University, Taoyuan City 320317, Taiwan; kuochen@cycu.edu.tw; 8 Ateneo Laboratory for Intelligent Visual Environments, Department of Information Systems and Computer Science, Ateneo de Manila University, Quezon City 1108, Philippines; pabu@ateneo.edu

**Keywords:** alveolar crest, cemento-enamel junction, Mask R-CNN, apical periodontitis, object detection

## Abstract

The severity of periodontitis can be analyzed by calculating the loss of alveolar crest (ALC) level and the level of bone loss between the tooth’s bone and the cemento-enamel junction (CEJ). However, dentists need to manually mark symptoms on periapical radiographs (PAs) to assess bone loss, a process that is both time-consuming and prone to errors. This study proposes the following new method that contributes to the evaluation of disease and reduces errors. Firstly, innovative periodontitis image enhancement methods are employed to improve PA image quality. Subsequently, single teeth can be accurately extracted from PA images by object detection with a maximum accuracy of 97.01%. An instance segmentation developed in this study accurately extracts regions of interest, enabling the generation of masks for tooth bone and tooth crown with accuracies of 93.48% and 96.95%. Finally, a novel detection algorithm is proposed to automatically mark the CEJ and ALC of symptomatic teeth, facilitating faster accurate assessment of bone loss severity by dentists. The PA image database used in this study, with the IRB number 02002030B0 provided by Chang Gung Medical Center, Taiwan, significantly reduces the time required for dental diagnosis and enhances healthcare quality through the techniques developed in this research.

## 1. Introduction

Periodontal disease is a common chronic inflammatory condition that often goes unnoticed by patients, causing them to miss the optimal treatment window [1,2,3]. When the alveolar bone is damaged, gingival recession and bone loss can expose the tooth roots or create sensitive teeth, compromising the stability of the teeth and potentially leading to tooth loss. If not treated promptly, the alveolar bone can also be affected, accumulating plaque [4,5]. Eventually, the atrophy causes periodontal pockets to expand, resulting in loose teeth. In such cases, early detection and treatment are crucial to prevent the condition from worsening and to maintain oral health.

Historically, dentists treating alveolar bone damage typically needed to perform alveolar bone surgery, whether to remove the damaged bone or to reshape it to its physiological form. This process required an assessment of alveolar bone loss, primarily relying on the judgment of the extent of erosion. The characteristics of this condition include the gradual destruction of the ALC [6], leading to periodontal pocket formation and gingival recession. The CEJ [7] refers to the anatomical structure where the enamel, which covers the dental crown, meets the cementum that coats the root [8]. It is a critical reference point in clinical dentistry, as it is generally the site where gingival fibers attach to a healthy tooth [9]. One of the primary parameters for evaluating periodontal destruction is the loss of connective tissue attachment to the tooth root surface. Consequently, the CEJ serves as a stable landmark for measuring clinical attachment loss and assessing periodontal damage [10]. Both PAs and bitewing radiographs have served as the standard for CEJ location assessment [11]. To assist dentists in more accurately diagnosing these conditions, this study utilized tooth recognition and segmentation techniques [12,13] to automate the identification of key locations such as the CEJ and the ALC. In current clinical practice, manual periapical detection faces several major challenges. Firstly, due to the low visibility of tooth gaps, the detection process is easily influenced by adjacent teeth, leading to erroneous judgments. Secondly, the varying angles of each tooth’s apex further increase the difficulty of detection, affecting the accuracy and reliability of the assessments. Due to the inconsistent angles of PA images compared to bitewing or panoramic radiographs, it is challenging to annotate these images conveniently [14]. Moreover, the variability in the quality of X-ray images further complicates the identification of the CEJ and ALC, necessitating the preprocessing of PA images [15] to enhance their quality.

As technology advances, the application of artificial intelligence (AI) has become increasingly widespread across various fields. For instance, endoscopic examinations [16], pancreatic cancer treatment [17], and lung nodules detection [18]. Significant progress has been made in the application of artificial intelligence in dentistry, surpassing the traditional methods that solely relied on visual inspection and professional expertise. Dentists can utilize image masking techniques to enhance the contrast of X-rays [19] for better determination of furcation [20]. The use of convolutional neural network (CNN) models for dental detection is increasing, such as for detecting maxillary sinusitis [21], identifying caries and restorations [22,23], accurately detecting implant positions, and assessing the damage caused by peri-implantitis [24] or dual-supervised network (DSN) models for tooth recognition and boundary regression [25]. In addition to the techniques, many studies have proposed improvements based on object detection models for medical image analysis [26], such as FLM-RCNN [27], the Levenberg–Marquardt backpropagation training algorithm [28], YOLO-DENTAL [29], and using feedforward neural networks for classifying lesions [30]. Various techniques have been applied to DPR images for detecting apical lesions [31] and inferior alveolar nerve injuries [32].

From the above, AI technology can effectively reduce the burden of dentists during consultations. Thus, this study utilizes both YOLO (You Only Look Once) and Mask R-CNN training models, combined with the preprocessing of PA images to enhance the accuracy of YOLO in detecting individual teeth. The Mask R-CNN model is applied to mask these images, specifically to identify and extract the CEJ and ALC positions.

## 2. Materials and Methods

This section will describe the methods and is divided into the following parts.

### 2.1. Study Design

**Figure 1:**
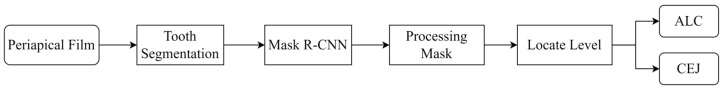
The overall flowchart in this research.

To assist dentists in more accurately diagnosing these conditions, this study utilized tooth detection and instance segmentation techniques to identify the location of the CEJ and the ALC automatically. Initially, PA images are preprocessed to enhance image quality, making the contours of teeth and alveolar bone clearer. Then, the YOLOv8 model is used to predict the position of individual teeth and, based on the localization results, the individual teeth are segmented from the PA images. Data augmentation techniques such as rotation angles are employed to increase the sample size of the database and prevent model overfitting. The segmented tooth images are classified, and the YOLOv8 classification model [33] excludes teeth that cannot be assessed, such as those with partially obscured CEJ levels or with implants and crowns. Subsequently, using the Detectron2 framework with Mask R-CNN, masks for the teeth, bone, and crown are extracted. These masks are used to determine the ALC level and CEJ level. The overall flowchart of this study is illustrated in Figure 1. The contributions of this study are as follows:

1. YOLOv8 achieved a sensitivity of 94.3% in extracting single teeth from original images. With the developed CLAHE algorithm, the sensitivity improved to 97.5%.
2. Mask R-CNN was utilized to extract three types of masks, with the DSC and Jaccard index both exceeding 90%. Additionally, the image augmentation method developed in this study showed an improvement of 1~3%.
3. A localization algorithm is proposed for CEJ and ALC; the RMSE is lower than 0.09, providing visualization techniques to aid dentists in diagnosis.

### 2.2. Data Collection

The database used in this study is sourced from the Chang Gung Memorial Hospital in Taoyuan, Taiwan. It was compiled by five dental practitioners, each with over five years of experience. The study received approval from the Institutional Review Board (IRB: 202301730B0), ensuring that ethical standards were met in the research process. To observe lesions near the alveolar bone, the use of PA imaging is specifically justified. Due to the larger scale of PA images, it is difficult to clearly observe localized lesions, making PA imaging a preferred choice in clinical practice. The smaller size of the PA image allows for more detailed local observation, thereby enhancing diagnostic accuracy. The selection of image sizes, such as 825×1200 and 1200×825, is based on providing more suitable proportions when sectioning teeth.

### 2.3. Statistical Analysis

The CEJ is an anatomical landmark on the tooth that separates the crown from the root structure, where the formation of enamel (the outermost layer of the crown) stops and the formation of cementum (the outermost layer of the root) begins [34]. This study references a technique [35] for lesion marking, serving as a basis for researchers. Dentists’ manually annotated results are exported to an Excel file, containing key point information marked on the images. These data serve as a baseline for comparison with the automated annotation results. The automated annotation system can detect the coordinates of the CEJ and ALC and compare them with the coordinates marked by dentists. To evaluate the accuracy of the automated annotation system, this study calculates the directional offset and further computes the root mean square error (RMSE). The RMSE quantifies the difference between the predicted and actual values, providing an assessment of the accuracy of the automated annotation system relative to the manual annotation system. This comparison helps understand the performance of the automated annotation method and its feasibility in practical applications.

### 2.4. Tooth Segmentation

**Figure 2:**
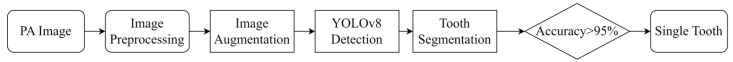
Tooth segmentation flowchart.

The steps involved in tooth segmentation in this study are illustrated in Figure 2. First, PA images are preprocessed to enhance model detection accuracy and facilitate annotation. These preprocessing steps include image enhancement, contrast adjustment, and noise reduction, ensuring that the contours of the teeth and alveolar bone are clearly visible. Next, the preprocessed PA images are annotated, specifically marking the locations of individual teeth. These annotated image data are used to train the YOLOv8 model, enabling it to accurately detect the positions of individual teeth. Once training is completed, the model is used to predict and automatically segment individual teeth from new images using the segment algorithm.

#### 2.4.1. PA Image Preprocessing

**Figure 3:**
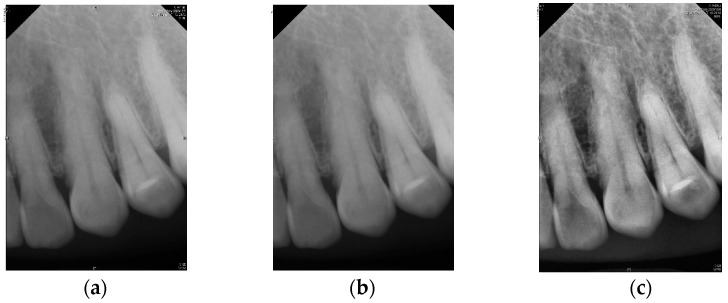
Image preprocessing in tooth segmentation steps: (**a**) original image, (**b**) median blur process, (**c**) CLAHE process.

The quality of the original image can affect the physician’s diagnosis. To clarify the contours of the teeth, this study preprocesses PA images to improve the detection accuracy of YOLOv8 and provide more precise annotations. First, the original image, Figure 3a, undergoes denoising using a median filter to remove extraneous noise, resulting in Figure 3b. This allows the noise to blend into the surrounding pixels, which is crucial because any remaining noise might be amplified during the subsequent CLAHE processing, thereby degrading image quality. Next, CLAHE is applied for preprocessing. This step enhances the contours of various objects in the image, such as the teeth, alveolar bone, and roots, making them more distinct, as shown in Figure 3c. A notable advantage of using CLAHE is that it enhances contrast based on local regions of the image rather than the global contrast, preventing issues where some parts of the image become too bright while others become too dark. These preprocessing steps are highly beneficial for the model training in this study and significantly improve accuracy.

#### 2.4.2. PA Image Annotation and Dataset Augmentation

**Figure 4:**
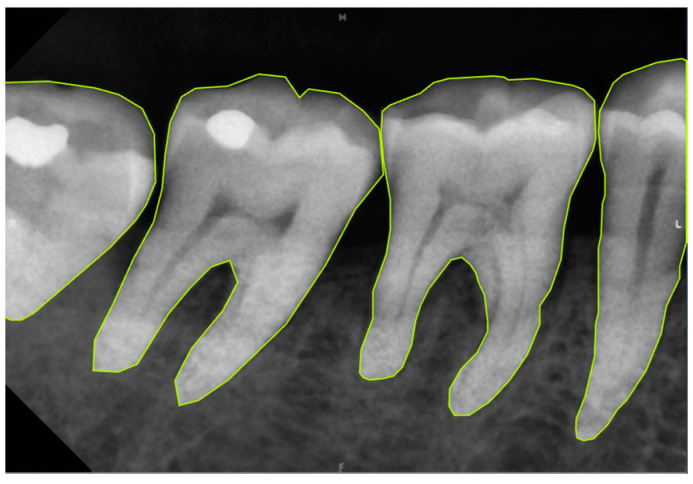
Image annotation example in YOLO object detection.

The Roboflow annotate tool was used to perform polygonal annotation on the processed teeth, as shown in Figure 4, and the annotation results were exported in TXT format. This step aims to construct the labeled dataset required for training the model. Data augmentations were employed to enhance the training effectiveness, including flipping the images and rotating 90° clockwise and counterclockwise, the YOLOv8 dataset detailed is shown in Table 1. YOLOv8, like its predecessors, is designed for real-time object detection. It includes innovations in the backbone, neck, and head of the network to enhance feature extraction and object detection accuracy. The model is trained using a loss function that combines classification loss, localization loss (bounding box regression), and confidence loss. Hyperparameters such as learning rate, batch size, number of epochs, and others need to be set. During the training phase, the performance of the model is evaluated at each epoch using the validation set. Based on the validation results, adjustments can be made to the model architecture, hyperparameters, or training process to improve performance. These data augmentation methods increase the diversity of the dataset, helping the model to capture the features of the teeth precisely. By integrating various preprocessing and data augmentation methods, the accuracy and generalization ability of the model in the tooth segmentation task can be significantly improved.

#### 2.4.3. YOLOv8 Detection

YOLO is a deep learning technique specifically designed for object detection, it uses a single forward propagation step to directly predict the positions and categories of all objects in an image. This allows YOLO to perform object detection efficiently in real-time while maintaining a high level of accuracy. This study uses the YOLOv8 model to predict the teeth’s positions and then segments individual teeth based on the model’s localization. Additionally, it compares the performance of YOLOv5, YOLOv7, and YOLOv8 in handling the tooth segmentation task. Initially, data preprocessing and augmentation are conducted to ensure that the training data are consistent and diverse, thereby enhancing the model’s generalization ability. The processed data are divided into training, validation, and test sets, as shown in Table 1 to facilitate model training and evaluation. Subsequently, the prepared datasets are input into the YOLOv5, YOLOv7, and YOLOv8 models for training, allowing the observation of their learning effectiveness and convergence rates; the indicators are calculated as follows, and the formula is shown in Equations (1)–(3). Table 2 provides a detailed overview of the hardware and software configurations used in the system. The hardware components include an AMD Ryzen 7 3700X CPU, an NVIDIA GeForce RTX 3070 GPU with 8 GB of memory, and 32 GB of DRAM. The software stack includes Python version 3.11.9, PyTorch version 2.4.0, and CUDA version 12.1.

(1) $$Precision=\frac{T_{p}}{T_{p}+F_{p}}$$

(2) $$Recall\left(Sensitivity\right)=\frac{T_{p}}{T_{p}+F_{n}}$$

(3) $$mAP=\frac{\sum_{q=1}^{Q}\;\frac{1}{n}\sum_{\;}^{\;}\;P_{n}\;\left(n=1,\;2,\;3,\;\ldots .\;,\;m\right)}{Q}$$

### 2.5. Mask R-CNN

**Figure 5:**
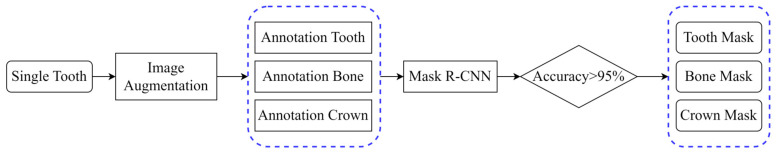
Mask R-CNN flowchart.

The individual single-tooth image extracted from the previous steps is input into the Mask R-CNN model, as shown in the workflow diagram in Figure 5. In this process, all teeth in the image are annotated, regardless of whether they are intact. This step aims to generate three types of masks, specifically for the tooth, bone, and crown, serving as the intermediate masked images for subsequent steps. These masked images will be used for further analysis and diagnosis, aiding in the precise localization and identification of dental structure, and enhancing the accuracy and reliability of the diagnosis.

#### 2.5.1. Single Tooth Image Augmentation

To improve accuracy and training effectiveness, this study applies vertical flipping to the single-tooth images obtained after CLAHE enhancement. This augmentation increases the number of single-tooth images, preventing overfitting of the Mask R-CNN model and providing more training data to enhance accuracy. Consequently, the training and validation datasets for this study have been doubled in size, as shown in Table 3, ensuring a more robust and comprehensive dataset for model training.

#### 2.5.2. Single-Tooth Annotation Mask

**Figure 6:**
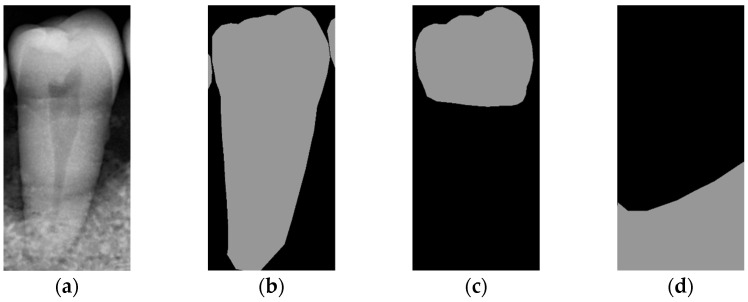
The three types of masks used in this study are (**a**) CLAHE tooth segmentation, (**b**) tooth annotation mask, (**c**) crown annotation mask, and (**d**) bone annotation mask.

To locate the ALC level and assist dentists in preliminarily identifying critical treatment areas, this study annotates the tooth bone and trains the Mask R-CNN model to extract the Bone Mask. For locating the CEJ level, the study annotates the tooth crown to extract the Crown Mask. The methods and processes for these annotations are illustrated in Figure 6. These steps are designed to accurately pinpoint the key anatomical structures of the teeth, enhancing the precision of dental diagnostics.

#### 2.5.3. Mask R-CNN

This study utilized the Detectron2 framework for mask extraction from dental images, opting for the Mask R-CNN model to achieve precise pixel-level segmentation. Mask R-CNN extends Faster R-CNN’s object detection results to perform pixel-level classification, with its main components including the backbone, head, and ROIAlign. ResNet-50 is used as the backbone to extract multi-level features from the images and generate candidate regions. The head component employs a Feature Pyramid Network (FPN) for classifying candidate regions and bounding box regression, producing the final detection results and pixel-level masks. Additionally, the ROIAlign component in Mask R-CNN precisely aligns the features of each candidate region on the feature map using bilinear interpolation, overcoming the quantization errors that may arise from traditional ROI pooling methods. This study employed Mask R-CNN to extract three distinct categories of masks required for the subsequent stages of the process. Initially, individual teeth were segmented, followed by the use of Mask R-CNN for mask extraction. This strategy narrows the detection scope of the model, which not only reduces the training time but also enhances the overall training process, leading to superior mask extraction results. During this phase, the data were divided approximately in a 7:3 ratio, with 140 images used for training and 54 for validation. Additionally, the dataset was augmented by vertically flipping the tooth images, effectively increasing the dataset size to 280 images for training and 108 for validation.

### 2.6. ALC Level Localization

The largest area of the Tooth Mask is retained and overlaid with the Bone Mask to form a composite mask. Subsequently, the custom localization algorithm developed in this study is applied to identify the ALC level for each symptomatic tooth in the PA image. This approach allows for the accurate identification of critical symptomatic regions, providing dentists with precise diagnostic information.

#### 2.6.1. Retained the Largest Mask

**Figure 7:**
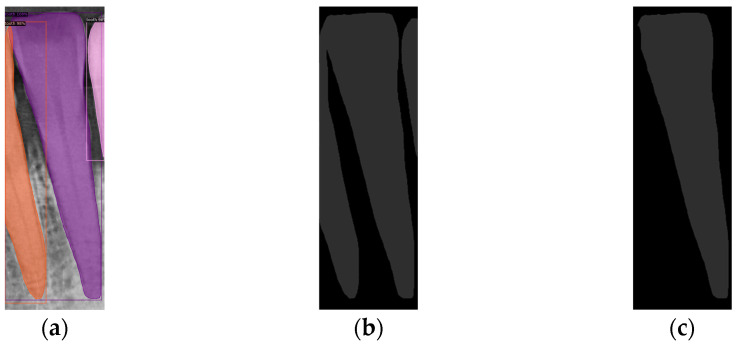
Mask R-CNN predictions for single tooth and Tooth Mask processing. (**a**) Mask R-CNN prediction. (**b**) The mask predicts the result. (**c**) Removing incomplete Tooth Mask.

Three different mask categories are obtained from the Mask R-CNN prediction results: Tooth Mask, Bone Mask, and Crown Mask. After acquiring these masks, only the fully segmented teeth are analyzed. For the incomplete teeth within the Tooth Mask, the neighboring Tooth Mask needs to be addressed. Since YOLOv8 was used earlier to extract individual teeth, each Tooth Mask contains one complete tooth, covering most of the area, allowing the removal of the masks of any incomplete teeth, as shown in Figure 7a,b. 

Instance segmentation not only requires detecting the object’s class and location but also involves pixel-level segmentation for each object, each object is segmented into a unique region, even if they belong to the same class. The study identifies all pixel values within the Tooth Mask and saves the masks predicted by Mask R-CNN, representing the pixels of the same object with the same value. The number of different pixel values is then calculated and the pixel value with the largest area is recorded. Other objects with different pixel values are removed, as shown in Figure 7c. This method ensures that only the complete Tooth Mask is used for analysis, thereby improving diagnostic accuracy and efficiency.

#### 2.6.2. Overlay and ALC Localization 

**Figure 8:**
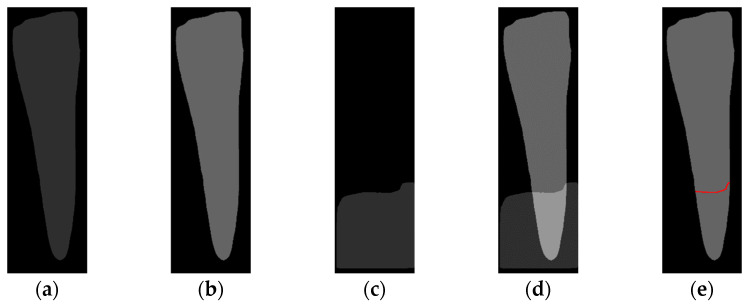
Mask processing in the ALC level localization algorithm. (**a**) Tooth Mask without new value. (**b**) Tooth Mask with new value. (**c**) Bone Mask. (**d**) Overlay. (**e**) ALC level localization (red line).

To locate the ALC level, the Bone Mask and Tooth Mask predicted by Mask R-CNN are overlaid. By analyzing the overlapping regions of these two masks, the position of the ALC level can be determined. The overlay process is illustrated in Figure 8. Since the teeth and bone might share the same pixel values, the pixel value differences cannot be used for localization. Therefore, this study assigns new pixel values to the Tooth Mask, as shown in Figure 8b, and uses these new pixel value differences to locate the ALC level. Next, a 5 × 5 kernel is iteratively applied across the entire image. If the kernel in a given iteration contains the pixel values from Figure 8b and the overlapping pixel values from Figure 8c, the kernel at that iteration is identified as the ALC level, as shown in Figure 8e.

### 2.7. CEJ Level Localization

The largest area of the Tooth Mask is retained, and the Crown Mask is subjected to dilation. The processed Tooth Mask and Crown Mask are then overlaid to form a composite mask. The developed localization algorithm is used to identify the CEJ position for each symptomatic tooth in the PA image. This method ensures precise localization by accurately identifying and processing key areas, improving diagnostic accuracy.

#### 2.7.1. Crown Mask Dilation

CEJ refers to the junction between the crown and root. It can be analyzed by overlaying the Tooth Mask and Crown Mask. Since it is not feasible to predict a Crown Mask that perfectly aligns with the crown position in the Tooth Mask, this leads to discrepancies in pixel locations and reduces the accuracy of localization. Hence, the Crown Mask can be dilated using Equation (4), ensuring that the crown completely envelops the crown portion of the Tooth Mask. This leaves only the necessary pixel discrepancy locations.

(4) $$Dilatation\left(A,\;B\right)=A⨁B$$

#### 2.7.2. Overlay and CEJ Localization 

**Figure 9:**
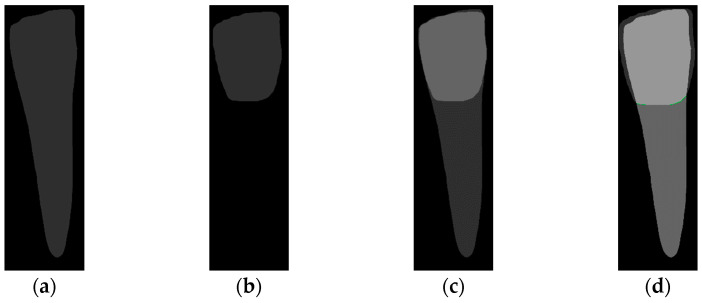
Mask processing in the CEJ level localization algorithm. (**a**) Tooth Mask. (**b**) Crown Mask without dilation. (**c**) Overlay with dilation. (**d**) CEJ level localization.

Due to the gingiva attached above the CEJ and its position at the junction between the crown and the root, this junction often becomes an error-prone area in symptom diagnosis. To help dentists make more precise and faster judgments or treatments when extracting symptoms, this study proposes a new method to identify the CEJ level. This technique involves overlaying the Tooth Mask and the dilated Crown Mask, allowing it to encompass the crown portion of the Tooth Mask. This method significantly enhances the accuracy of CEJ localization, as demonstrated in Figure 9d.

**Figure 10:**
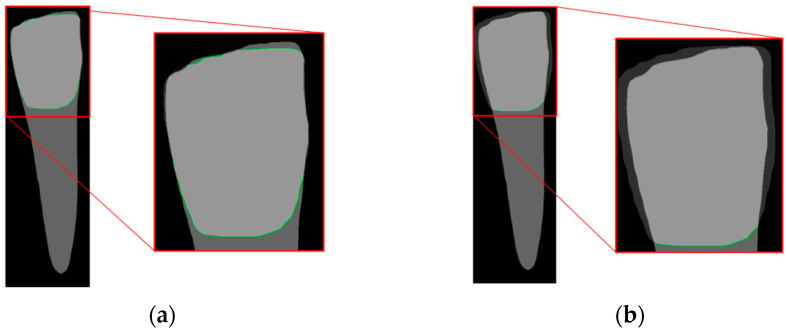
Magnified comparison of Crown Mask (**a**) without dilation and (**b**) with dilation.

Figure 10 compares the effectiveness of the CEJ level localization algorithm with and without the dilation process in this study. Initially, the analysis of the un-dilated Crown Mask revealed that the top of the crown often had many overlapping masks, which hindered the accurate localization of the CEJ level. Therefore, after applying the dilation process to the Crown Mask, the localization results improved significantly, ensuring that the mask intersection occurred only at the junction between the crown and the root. This approach markedly enhances the accuracy of CEJ level localization, thereby better aiding in the diagnosis and treatment of symptoms.

## 3. Results

In this chapter, this study will analyze the experimental results and compare them with advanced research. This chapter can be divided into tooth detection, different mask evaluation, and the CEJ/ALC position result.

### 3.1. Tooth Detection Result

The YOLO detection training results, presented in Table 4, highlight the superior performance of YOLOv8 compared to YOLOv5 and YOLOv7. Training with image enhancement significantly improved YOLOv8’s precision from 0.943 to 0.978, recall from 0.943 to 0.975, mAP50 from 0.941 to 0.989, and mAP50-90 from 0.805 to 0.855. Comparatively, YOLOv5 achieved a precision of 0.930 on original images and 0.959 on enhanced images, while YOLOv7 reached 0.939 and 0.969, respectively. These results demonstrate that YOLOv8, particularly with image enhancement, excels in detecting individual teeth, confirming the effectiveness of data augmentation in improving performance.

After training the YOLOv8 detection model, this study used an untrained test dataset for accuracy prediction and evaluated accuracy through the confusion matrix, as shown in Equation (5). The validation results are presented in Table 5, where the accuracy of the YOLOv8 detection model with CLAHE enhancement reached 97.01%, significantly higher than YOLOv7’s 95.37% and YOLOv5’s 90.59%. This indicates a notable improvement of the YOLOv8 model over its predecessors.

(5) $$Accuracy=\frac{T_{P}+F_{P}}{T_{P}+F_{P}+T_{N}+F_{N}}$$

This study implemented additional preprocessing on the images after thorough consideration, resulting in a significant improvement in accuracy and further validating the reliability of the model. According to the training process outlined in Table 6, each set of 20 epochs took approximately one and a half minutes. The model’s loss rate demonstrated an exponential decline, indicating the overall effectiveness of the training. This decline in loss rate can serve as a key indicator of the model’s training status.

**Figure 11:**
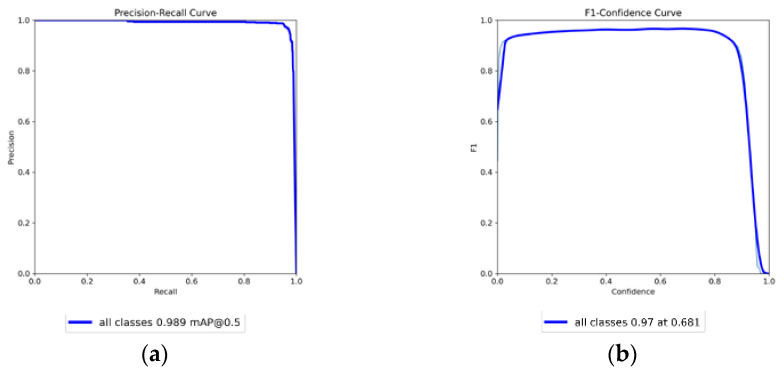
Model performance and training metrics: (**a**) Precision–Recall curve and (**b**) F1–Confidence curve.

The YOLOv8 model was used to predict the unmarked and untrained original test dataset, with the prediction results shown in Table 7. The evaluation was conducted using the Precision–Recall curve, as shown in Figure 11. The Precision–Recall curve illustrates the model’s precision and recall at different IOU thresholds. In Figure 11a, when the IOU equals 0.5, the mAP reaches 0.989. Additionally, the closer the curve is to the upper right corner of the coordinate axis, the better the model’s performance in correctly identifying samples. The F1–Confidence curve shows the variation of the F1 score at different confidence thresholds, with the highest F1-score of 0.97. The Precision–Recall curve of this study is very close to the upper right corner, indicating that the model has high precision and high recall.

### 3.2. Tooth, Crown, and Bone Mask Result

Table 8 shows the comparison of the Dice Similarity Coefficient (DSC) and Jaccard index for Tooth Mask, Bone Mask, and CEJ line with and without image enhancement. When classifying the Tooth Mask using Mask R-CNN, the DSC for the original image was 0.9425, which improved to 0.9478 after enhancement; the Jaccard index increased from 0.8920 to 0.9015. For Bone Mask classification, the DSC for the original image was 0.9273, improving to 0.9352 with enhancement; the Jaccard index rose from 0.8688 to 0.8992. Notably, in the CEJ line classification, the DSC for the original image was 0.9500, which increased to 0.9550 after enhancement; the Jaccard index improved from 0.9071 to 0.9156. The Mask R-CNN model in this study showed higher accuracy in classifying the Tooth Mask, Bone Mask, and CEJ line after image enhancement, particularly making a notable contribution to CEJ line classification. This improvement is crucial for the detection and diagnosis of teeth and surrounding tissues. The Dice Similarity Coefficient (DSC) and Jaccard index formulae are shown in Equations (6) and (7); the DSC and Jaccard index are commonly used metrics for evaluating the similarity between two sets in mask-based models. In these models, the annotated masks and predicted results are typically treated as two sets, with pixel locations as the elements of the sets for computation. The DSC focuses on the overlapping region of the masks, assigning double weight to the overlap as indicated by its formula, making the DSC more sensitive to the overlapping area. Its values range from 0 to 1, with a higher value indicating a greater overlap between the masks and thus better model performance. In contrast, the Jaccard index evaluates the similarity of the overall sets by measuring the ratio of the intersection to the union of the masks. It also ranges from 0 to 1, with higher values reflecting the greater similarity of the overall sets. These two metrics offer different perspectives on measuring mask similarity, with the DSC emphasizing the importance of overlapping areas, while the Jaccard index focuses on the ratio of the entire sets.

(6) $$Dice\left(A,\;B\right)=\;\frac{2\;\left|A\;\cap \;B\right|}{\left|A\right|+\left|B\right|}$$

(7) $$Jaccard\;index\left(A,\;B\right)=\frac{\left|A\;\cap \;B\right|}{\left|A\;\cup \;B\right|}$$

**Figure 12:**
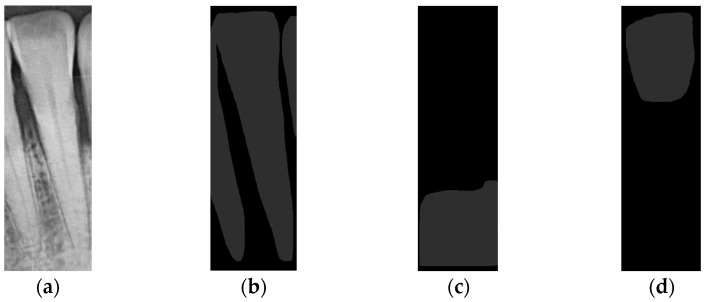
Mask R-CNN prediction result: (**a**) single tooth (CLAHE), (**b**) Tooth Mask, (**c**) Bone Mask, and (**d**) Crown Mask.

The prediction results of the three masks (Tooth, Bone, and Crown) using the aforementioned steps are shown in Figure 12, with different AP metrics evaluated using Equation (8). Table 9 presents the training results of Mask R-CNN for extracting these masks from original and enhanced images, assessed through AP, AP50, and AP75 metrics. For the Tooth Mask, image enhancement improved the AP from 66.73% to 69.65%, AP50 from 88.32% to 89.54%, and AP75 from 74.65% to 81.66%. For the Bone Mask, the AP increased from 73.32% to 76.66%, AP50 from 98.15% to 99.86%, and AP75 from 90.17% to 92.26%. For the Crown Mask, the AP improved from 79.14% to 81.55%, AP50 remained at 99.99%, and AP75 slightly decreased from 98.02% to 96.29%. These results indicate that whether using the bounding box or segmentation evaluation methods, the training outcomes for all three masks improved after image enhancement. This demonstrates the significant effectiveness of image enhancement techniques in increasing the detection accuracy of the Mask R-CNN model. The training process is shown in Table 10. The accuracy of Faster R-CNN for bounding box prediction and Mask R-CNN for mask segmentation prediction has significantly increased to more than 95% during the training process.

(8) $$AP=\int_{0}^{1}P\left(R\right)\;dR$$

### 3.3. CEJ and ALC Position Result

Table 11 presents the accuracy of the Mask R-CNN model for three categories, comparing the results from original images with those from enhanced images. The results show that image enhancement had the most significant improvement in the accuracy of the Tooth Mask, increasing from 92.63% to 93.48%. For the Bone Mask, the accuracy slightly improved from 95.50% to 96.95%. However, for the Crown Mask, the accuracy slightly decreased from 96.79% to 96.21% after image enhancement. This indicates that while image enhancement generally has a positive impact on the model’s accuracy, its effects can vary across different mask categories. Table 12 provides a comparative analysis between our developed positioning algorithm and the annotations supplied by the dentist. The upper portion demonstrates the CEJ and ALC levels ascertained by the algorithm, juxtaposed with the dentist’s marked points on both the left and right sides. Specifically, these points encompass CEJ (left) and CEJ (right) for the CEJ level and ALC (left) and ALC (right) for the ALC level. The lower segment portrays a segmented image, showcasing the positions identified by the algorithm in contrast to those identified by the dentist. The blue and purple circles represent the annotations made by the dentist and the red and green circles represent the annotation result of this study. Furthermore, it computes precision in every CEJ and ALC point on both sides, along with determining the Root Mean Square Error (RMSE) between the four points identified by the algorithm and the dentist’s annotations; the RMSE value between different points is lower than 0.09, and the minimum value is 0.0209 in CEJ (right). The RMSE formula is shown in Equation (9).

(9) $$RMSE=\sqrt{\sum_{i=1}^{n}\frac{{\left({\hat{y}}_{i}-y_{i}\right)}^{2}}{n}}$$

## 4. Discussion

This study evaluated the efficacy of using advanced image processing and deep learning techniques for periodontal diagnosis. The primary objective was to accurately detect and segment individual teeth and key anatomical landmarks, such as the CEJ and ALC, using PA imaging. Initially, the PA images underwent preprocessing with CLAHE to enhance image quality shown in Figure 3. This preprocessing significantly clarified the contours of teeth and alveolar bone, facilitating more precise annotations and model training. This study utilized the YOLOv8 and Mask R-CNN models to predict and segment dental images and applied image enhancement techniques to improve the accuracy of these models. For tooth detection, the YOLOv8 model was employed. The model achieved a sensitivity of 94.3% on the original images, which improved to 97.5% after applying the CLAHE enhancement. YOLOv8 demonstrated superior performance compared to its predecessors, YOLOv5 and YOLOv7, particularly in terms of precision, recall, mAP, and detection accuracy. The enhanced YOLOv8 model reached an impressive accuracy of 97.01% on the test dataset, as shown in Table 13. The study also conducted a detailed comparison with the methods in [36,37]. The approach in [36] uses a matrix to calculate inter-proximal space for tooth segmentation, relying heavily on matrix operations to locate these spaces, which works well when teeth are closely aligned. However, its accuracy may diminish with irregular or overlapping teeth. Meanwhile, the algorithm proposed by Nomir and Abdel-Mottaleb in [37] achieves tooth segmentation by separating teeth from the background using horizontal and vertical projections. This method performs well with images where teeth and background contrast sharply, but its accuracy can decrease in complex or low-contrast backgrounds.

According to the training results shown in Table 9, image enhancement significantly improved the performance of the Mask R-CNN model. For instance, for the Tooth Mask, the AP increased from 66.73% to 69.65%, AP50 from 88.32% to 89.54%, and AP75 from 74.65% to 81.66% after image augmentation. For the Bone Mask, the AP rose from 73.32% to 76.66%, AP50 from 98.15% to 99.86%, and AP75 from 90.17% to 92.26%. Regarding the Crown Mask, the AP increased from 79.14% to 81.55%, AP50 remained at 100%, and AP75 slightly decreased from 98.02% to 96.29%. These results indicate that the training outcomes for all three mask categories improved after image enhancement, demonstrating the significant effectiveness of image enhancement techniques in increasing the detection accuracy of the Mask R-CNN model. Additionally, Table 11 shows the accuracy comparison of the Mask R-CNN model for the three mask categories. The results indicate that image enhancement had the most significant improvement in the accuracy of the Tooth Mask, increasing from 92.63% to 93.48%. For the Bone Mask, the accuracy slightly improved from 95.50% to 96.95%. However, the accuracy of the Crown Mask slightly decreased from 96.79% to 96.21% after image enhancement. This suggests that while image enhancement generally positively impacts the model’s accuracy, its effects can vary across different mask categories. In contrast, the Unet model performed lower in both the DSC and Jaccard index across all classifications [6]; the comparison is shown in Table 14. This highlights the significant advantage of the Mask R-CNN model in handling CEJ line classification. Another significant contribution of this study is the new method proposed for locating the CEJ and ALC levels, which can be seen in Table 12. Throughout Mask R-CNN and image processing to evaluate ALC and CEJ position, the last RMSE is 0.0209 at CEJ (right). These localization techniques assist dentists in faster and more accurate symptom evaluation, significantly enhancing diagnostic efficiency and precision.

Specifically, the developed image enhancement and mask segmentation techniques in this study significantly improved the classification accuracy of the CEJ line, providing robust support for the early diagnosis and treatment of periodontitis. This study has several limitations. First, variations in image quality could impact the model’s accuracy, especially in clinical applications where low-quality or noisy images are more common. Additionally, the reliance on manual annotations introduces potential biases; even experienced dentists may have inconsistencies in their annotations, which can influence the training outcomes of the model. While YOLOv8 and Mask R-CNN performed well in this study, these models may face challenges in handling more complex cases, such as overlapping or missing teeth and severe periodontal diseases. The practical implementation of these AI technologies in clinical settings also necessitates seamless integration into existing diagnostic workflows and ensuring that practitioners can effectively utilize these tools. Future research will focus on exploring various periodontal conditions, including calculating bone loss to assist clinicians in quickly assessing patients’ conditions. The study will emphasize optimizing algorithms for the localization of the CEJ and the ALC, aiming to enhance the system’s efficiency and accuracy. Additionally, different object detection and semantic segmentation models will be compared to identify the most suitable model for PA images, which will be integrated into future systems. The research will also concentrate on utilizing algorithms to aid clinicians in calculating bone loss and addressing other periodontal conditions.

## 5. Conclusions

The primary objective of this study was to accurately locate the CEJ and ALC to improve the accuracy and efficiency of periodontal diagnosis. By employing CLAHE technology for image preprocessing and utilizing the YOLOv8 and Mask R-CNN models for tooth detection and region segmentation, we significantly enhanced the accuracy of automated assessments. The study results demonstrate that these innovative techniques effectively reduce the time required for diagnosis while increasing accuracy, which holds significant implications for dental practitioners. Specifically, this research provides a rapid and reliable method for marking and assessing symptoms in dental X-rays, aiding dentists in better diagnosing and treating periodontal diseases. This study not only highlights the potential of AI technology in dentistry but also offers a practical tool for clinical practice, significantly improving diagnostic efficiency and accuracy.

## Figures and Tables

**Table 1:** YOLOv8 detection dataset.

	Train	Validation	Test
Original	140	57	84
Dataset Augmentation	420	171	

**Table 2:** The hardware and software platform vision.

Hardware Platform	Version	Manufacturer	
CPU	AMD Ryzen 7 3700X	Intel, California, United States	
GPU	NVIDIA GeForce RTX 3070 8 G	NVIDIA, California, United States	
DRAM	32 GB	ADATA, New Taipei City, Taiwan	
Software Platform	Version	Software Platform	Version
Python	3.11.9	Anaconda	24.1.2
PyTorch	2.4.0	CUDA	12.1

**Table 3:** Mask R-CNN train and validation dataset.

	Train	Validation
Original	140	54
Image Augmentation	280	108

**Table 4:** YOLO detection result.

		Precision	Recall	mAP50	mAP50-90
YOLOv5	Original	0.930	0.950	0.942	0.787
	CLAHE	0.959	0.977	0.979	0.847
YOLOv7	Original	0.939	0.947	0.948	0.802
	CLAHE	0.969	0.960	0.983	0.828
YOLOv8	Original	0.943	0.943	0.941	0.805
	CLAHE	0.978	0.975	0.989	0.855

**Table 5:** Tooth detection training accuracy.

Method	Accuracy	
YOLOv5	Original	84.50%
	CLAHE	90.59%
YOLOv7	Original	93.23%
	CLAHE	95.37%
YOLOv8	Original	95.44%
	CLAHE	97.01%

**Table 6:** YOLOv8 segmentation training process in every 20 epochs.

Epoch	Time Elapsed	Segmentation Loss
1	00:00:05	2.52
21	00:01:33	1.38
41	00:03:03	1.23
61	00:04:14	1.13
81	00:05:34	1.00
100	00:06:57	0.77

**Table 7:** Single tooth segmentation validation.

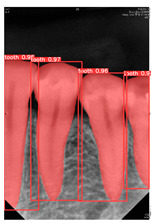	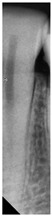	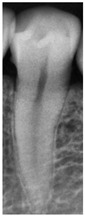	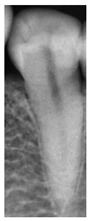	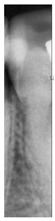
(a)	(b)	(c)	(d)	(e)
Accuracy	96.51%	97.12%	96.46%	93.77%

**Table 8:** Comparison of DSC and Jaccard index for Tooth Mask and Bone Mask.

	Mask Category	DSC		Jaccard Index	
		Original	Augmentation	Original	Augmentation
This study	Tooth Mask	0.9425	0.9478	0.8920	0.9015
	Bone Mask	0.9273	0.9352	0.8688	0.8992

**Table 9:** Training results of Mask R-CNN for extracting tooth, bone, and crown (AP, AP50, AP75) with original and image augmentation.

Category	Evaluation Method	AP		AP50		AP75	
		Original	Augmentation	Original	Augmentation	Original	Augmentation
Tooth Mask	Bounding Box	66.73	69.65	88.32	89.54	74.65	81.66
	Segmentation	65.79	67.71	89.31	90.77	77.34	79.90
Bone Mask	Bounding Box	73.32	76.66	98.15	99.86	90.17	92.26
	Segmentation	70.55	72.19	98.15	99.86	80.12	83.15
Crown Mask	Bounding Box	79.14	81.55	99.99	99.99	98.02	96.29
	Segmentation	83.86	85.57	99.99	99.99	98.02	98.00

**Table 10:** Training process with Crown Mask and image augmentation over 1000 iterations.

Iteration	Fast R-CNN Accuracy	Mask R-CNN Accuracy	Total Loss
20	7.42%	62.29%	2.69
200	92.57%	80.65%	0.85
400	96.87%	92.93%	0.50
600	98.24%	95.89%	0.28
800	98.73%	96.56%	0.22
1000	98.73%	96.21%	0.21

**Table 11:** Comparison between different mask categories in the Mask R-CNN model.

	Mask Category	Original	Image Augmentation
Accuracy	Tooth Mask	92.63%	93.48%
	Bone Mask	95.50%	96.95%
	Crown Mask	96.79%	96.21%

**Table 12:** Comparison of the CEJ and ALC positioning algorithm results with dentist annotations.

Ground Truth	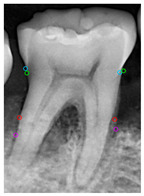			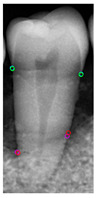		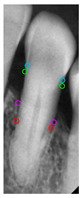		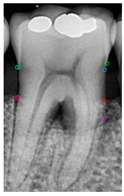		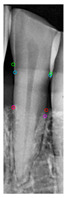
This Study	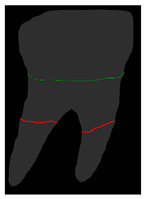			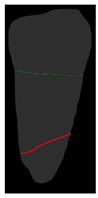		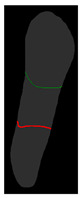		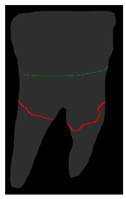		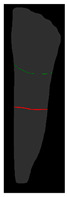
Number	No. 1			No. 2		No. 3		No. 4		No. 5
Precision										Average RMSE
		No. 1	No. 2		No. 3		No. 4		No. 5	
CEJ (left)		90.56%	95.39%		85.52%		90.35%		81.18%	0.0420
CEJ (right)		96.05%	99.10%		92.37%		92.39%		97.14%	0.0209
ALC (left)		85.65%	92.83%		81.48%		89.40%		99.11%	0.0843
ALC (right)		92.12%	96.94%		92.82%		97.52%		93.67%	0.0552

**Table 13:** Comparison with different object detection methods.

YOLOv8	Original	95.44%
	CLAHE	97.01%
Compared with [36]		79.60%
Compared with [37]		82.50%

**Table 14:** Comparison with different method in Tooth Mask and Bone Mask.

Method	Mask Category	DSC	Jaccard Index
This study	Tooth Mask	0.9478	0.9015
	Bone Mask	0.9352	0.8992
Ref. [6] (Unet)	Tooth Mask	0.8994	0.8225
	Bone Mask	0.8621	0.9319
Ref. [6] (ResNet-34 Encoder)	Tooth Mask	0.9170	0.9143
	Bone Mask	0.9235	0.9343

## Data Availability

The original contributions presented in the study are included in the article, further inquiries can be directed to the corresponding authors.
